# A network perspective on the topological importance of enzymes and their phylogenetic conservation

**DOI:** 10.1186/1471-2105-8-121

**Published:** 2007-04-11

**Authors:** Wei-chung Liu, Wen-hsien Lin, Andrew J Davis, Ferenc Jordán, Hsih-te Yang, Ming-jing Hwang

**Affiliations:** 1 Institute of Biomedical Sciences, Academia Sinica, Taipei 115, Taiwan; 2 Biochemistry Section, Max Planck Institute for Chemical Ecology, Hans-Knoell-Strasse 8, 07745 Jena, Germany; 3 Collegium Budapest, Institute for Advanced Study, Szentháromság utca 2, H-1014, Budapest, Hungary; 4 Animal Ecology Research Group, HAS, Hungarian Natural History Museum, Ludovika t. 2., H-1083, Budapest, Hungary

## Abstract

**Background:**

A metabolic network is the sum of all chemical transformations or reactions in the cell, with the metabolites being interconnected by enzyme-catalyzed reactions. Many enzymes exist in numerous species while others occur only in a few. We ask if there are relationships between the phylogenetic profile of an enzyme, or the number of different bacterial species that contain it, and its topological importance in the metabolic network. Our null hypothesis is that phylogenetic profile is independent of topological importance. To test our null hypothesis we constructed an enzyme network from the KEGG (Kyoto Encyclopedia of Genes and Genomes) database. We calculated three network indices of topological importance: the degree or the number of connections of a network node; closeness centrality, which measures how close a node is to others; and betweenness centrality measuring how frequently a node appears on all shortest paths between two other nodes.

**Results:**

Enzyme phylogenetic profile correlates best with betweenness centrality and also quite closely with degree, but poorly with closeness centrality. Both betweenness and closeness centralities are non-local measures of topological importance and it is intriguing that they have contrasting power of predicting phylogenetic profile in bacterial species. We speculate that redundancy in an enzyme network may be reflected by betweenness centrality but not by closeness centrality. We also discuss factors influencing the correlation between phylogenetic profile and topological importance.

**Conclusion:**

Our analysis falsifies the hypothesis that phylogenetic profile of enzymes is independent of enzyme network importance. Our results show that phylogenetic profile correlates better with degree and betweenness centrality, but less so with closeness centrality. Enzymes that occur in many bacterial species tend to be those that have high network importance. We speculate that this phenomenon originates in mechanisms driving network evolution. Closeness centrality reflects phylogenetic profile poorly. This is because metabolic networks often consist of distinct functional modules and some are not in the centre of the network. Enzymes in these peripheral parts of a network might be important for cell survival and should therefore occur in many bacterial species. They are, however, distant from other enzymes in the same network.

## Background

Enzymes are proteins that catalyze metabolic reactions vital for the survival and functioning of cells [1]. However, there is no reason to suppose that all enzymes have equal importance in metabolic pathways or that each enzyme occurs in all species. There is, in fact, great heterogeneity in the occurrence of particular enzymes within phylogenies [2]. Because enzymes are inherent parts of metabolic networks, one way to explore the reasons for this heterogeneity is to examine the network characteristics of the enzymes.

Many researchers have applied network analysis to various fields of molecular biology and have elucidated the organization and evolution of different molecular networks [3,4]. In several molecular networks, the number of connections of a network node tends to follow a power law distribution: most nodes have few connections and few nodes are well-connected to others [5-7]. These few well-connected nodes play important roles in their respective networks. In a metabolic network, a well-connected node is a metabolite acting as a hub, ensuring fast and efficient interconversions of metabolites [5]. Well-connected proteins or genes in protein-protein or genetic interaction networks seem to be vital for the survival of cells [6,7]. A power law distribution of nodal connections is likely to render a molecular network robust to random errors but vulnerable to targeted attacks [3,8].

One possible mechanism that might produce a power law distribution of links is if, during evolution, new nodes tend to attach preferentially to well connected network nodes [3,8].

An enzyme-enzyme relationship can be defined for two enzymes if the product of one is the substrate of the other [9,10]. It is therefore relatively easy to construct an enzyme-enzyme relation network (hereafter "enzyme network") from known metabolic reactions [9,10]. Recently, network analysis has been applied to enzyme or metabolic networks of specific organisms to reveal the underlying network structure [11] and to determine the phylogenetic distances between different species [12,13]. It has also been used to elucidate the mechanism of enzyme evolution[14,15] and to investigate the robustness of metabolic networks to damage such as enzyme deletion [16]. In this paper, we adopt a different approach by compiling all known enzyme-enzyme relations for a major group of organisms into an enzyme network. We also determine the phylogenetic profile for each enzyme (a term originally used by Pellegrini et al. [17]), which is the number of different species having a particular enzyme. We then determine the topological or positional importance of every individual enzyme in the enzyme network we had constructed. The specific null hypothesis we test is that the phylogenetic profile of enzymes is independent of their positional importance within the network. We examine whether enzymes with major topological roles occur more frequently (i.e. have higher phylogenetic profiles). This question can now be examined because recent advances in molecular biology have led to the production of metabolic pathway databases for hundreds of organisms [2,18]. A well-developed set of network indices is also now available that can quantify the topological importance of individual nodes[19,20] that have been used to reveal the connectivity structure and centrality of metabolic networks [21,22]. The metabolic databases provide information from which one can easily construct the enzyme network and determine the phylogenetic profile of enzymes. We use such information from a large database of metabolic pathways in bacterial species to test our null hypothesis. We also use several subsidiary tests to examine the characteristics of the local environments of enzymes. We then discuss possible reasons for the link between phylogenetic profile and topological importance, if such a link does indeed exist.

## Results and Discussion

**Figure 1:**
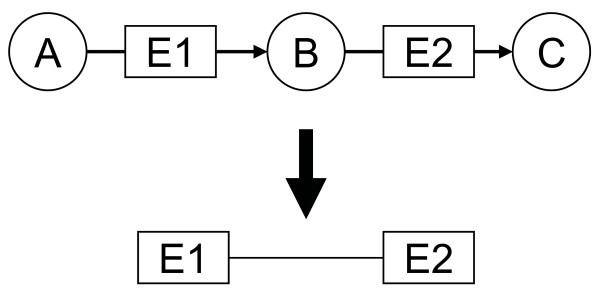
A schematic diagram illustrating the definition of a link between two enzymes. An enzyme-enzyme relation exists if two enzymes are involved in two successive reactions such that the product of one is the substrate of the other.

**Figure 2:**
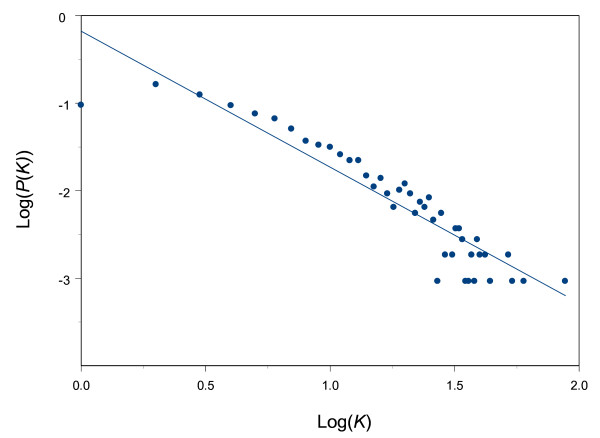
Distribution of the number of connections of nodes in the enzyme network with both axes plotted on log scales. A power-law, *P*(*k*)~*k*^-*γ*^, is fitted to the distribution with *γ *estimated to be 1.55 (*r*^2 ^= 0.862).

We constructed an enzyme network by combining information on all the enzyme-catalyzed metabolic reactions of 288 bacterial species from the KEGG database [2] (see Methods). In this network, a node represents an individual enzyme. A link between two enzymes exists if the product of one enzyme is the substrate of the other (Figure 1): we assume that reactions are reversible and therefore each link or enzyme-enzyme relation in the network is undirected. The enzyme network constructed from the KEGG database has 1081 nodes (i.e. enzymes) and 4169 links (i.e. enzyme-enzyme relation). As in many other molecular networks [3,5-8], the number of connections of a node tends to follow a power law distribution, *P*(*k*)~*k*^-*γ *^(i.e. a scale-free network), where *P*(*k*) is the probability that a node has *k *connections and *γ *is an exponent with an estimated value of 1.55 (*r*^2 ^= 0.862) for the network shown here (Figure 2). This suggests that most nodes in this enzyme network are sparsely connected while only a few have many connections to others. Networks with power-law distributions in their connectivity, or scale-free networks, are known to be robust against random errors or node deletion; in other words they are less likely to disintegrate into isolated parts when nodes are randomly removed from them [3,8].

### Correlation between phylogenetic profile and topological importance of enzymes

**Figure 3:**
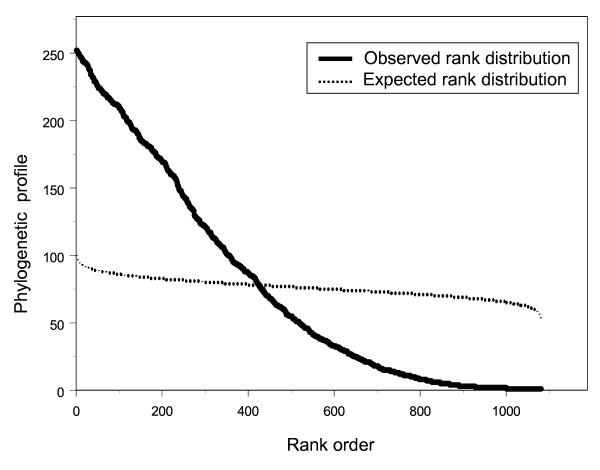
Ranked distribution of enzymes according to phylogenetic profile. The solid line represents the observed data from the KEGG database. The dotted line represents the expected phylogenetic profile for individual ranks (see Methods). The observed rank abundance distribution is significantly different from that expected (Χ^2 ^= 67299.55, *p *≈ 0, d.f. = 1080).

From KEGG we determined the phylogenetic profile (*F*) of every enzyme in the network. This is the number of the 288 bacterial species that contain a given enzyme. The value of *F *is extremely variable (Figure 3) with a range of 1–251. Many enzymes occur in only one bacterial species, but one enzyme occurs in 251 of the 288 species in the database.

For every enzyme in this network, we calculated three topological indices [19-22] (see Methods): "degree" (*D *– the number of direct neighbors a node has); "closeness centrality" (*C *– how close a node is to all others in the same network); and "betweenness centrality" (*B *– how frequently a node appears on all shortest paths between all pairs of nodes in the network). The Spearman rank correlations between the phylogenetic profile and each of the three measures of topological importance were all significant. This falsifies our null hypothesis that phylogenetic profile and measures of positional importance are independent. Enzymes that occur in more bacterial species tend to occupy more important topological positions in the network. The *F*-*B *and *F*-*D *correlations are positive but that of *F*-*C *is slightly negative. Of the three correlations, the strongest is between *F *and *B *(*r*_*F*, *B *_= 0.35, *p *≈ 0), followed by that between *F *and *D *(*r*_*F*, *D *_= 0.30, *p *≈ 0) and lastly, that between *F *and *C *(*r*_*F*, *C *_= -0.06, *p *= 0.03).

**Figure 4:**
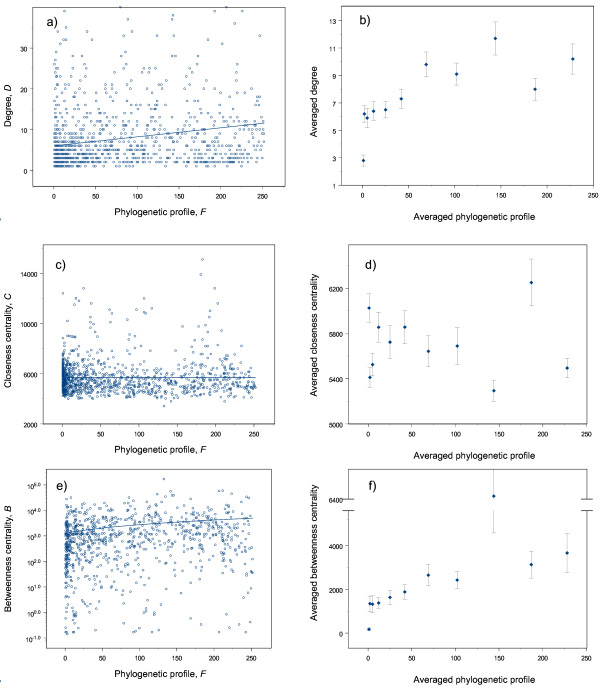
Scatter plots of phylogenetic profile against different measures of topological importance. (a) phylogenetic profile (*F*) against the degree (*D*); (b) averaged phylogenetic profile against averaged degree; (c) phylogenetic profile (*F*) against closeness centrality (*C*); (d) averaged phylogenetic profile against averaged closeness centrality; (e) phylogenetic profile (*F*) against betweenness centrality (*B*); (f) averaged phylogenetic profile against averaged betweenness centrality. The vertical bars in b, d, f are standard errors.

However, these relationships are very noisy, as demonstrated by the graphs of *F *against *D*, *C *and *B *(Figure 4a, 4c, 4e). Since our knowledge of metabolic pathways is not complete for each of the organisms examined, and other processes not accounted for by measures of topological importance might affect the phylogenetic profile, our results will contain a certain degree of inaccuracy or uncertainty. To reduce the noise and reveal more general relationships, we ranked the enzymes according to their phylogenetic profiles and divided them into groups of 100. For each group, we calculated the average phylogenetic profile and the corresponding average measure of topological importance. Using these averaged values, the rank correlations of phylogenetic profile with degree (*r*_*F*, *D *_= 0.91, *p *= 0.0042) and betweenness centrality (*r*_*F*, *B *_= 0.95, *p *= 0.0027) were again significant, and more evident; both these measures of topological importance tend to increase with phylogenetic profile (Figure 4b, 4f).

However, the correlation between phylogenetic profile and closeness centrality became insignificant (*r*_*F*, *C *_= -0.12, *p *= 0.68, Figure 4d). Similarly, when we divided the enzymes into groups of 50s, the rank correlations of phylogenetic profile with degree and betweenness centrality remained stronger and more evident than that with closeness centrality (*r*_*F*, *D *_= 0.84, *p *= 0.0001; *r*_*F*, *B *_= 0.83, *p *= 0.0001; *r*_*F*, *C *_= -0.065, *p *= 0.7641).

These results suggest that simple measures of topological importance can reveal interesting information about heterogeneities in the phylogenetic profile of enzymes. We found that betweenness is a good predictor of how many bacterial species have a particular enzyme, closely followed by degree. The relationship with closeness is much weaker or non-existent. The differences in predictive ability among these topological measures might reflect the structural organization of the enzyme network. Enzymes incident to many shortest paths between any other two enzymes (i.e. high betweenness) tend to occur in most bacterial species. This reflects the central role that such enzymes play in relaying metabolites from one enzyme to another. These enzymes can be considered positionally critical nodes; deleting them might compromise the integrity of the enzyme or metabolic network. Such enzymes should therefore occur in many bacterial species.

**Figure 5:**
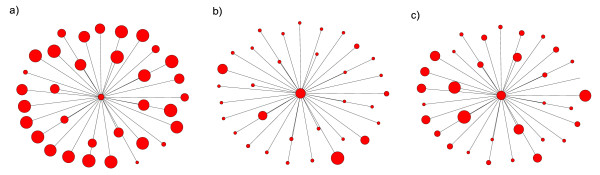
A sub-network consisting of enzyme EC2.7.1.69 and its direct neighbors. For simplicity, links between neighbors are omitted. Nodes represent enzymes, and links represent enzyme-enzyme relations between EC2.7.1.69 and its direct neighbors. The sizes of the nodes are proportional to (a) closeness centrality, (b) betweenness centrality and (c) phylogenetic profile. The networks were drawn using NETDRAW (Analytic Technologies).

**Figure 6:**
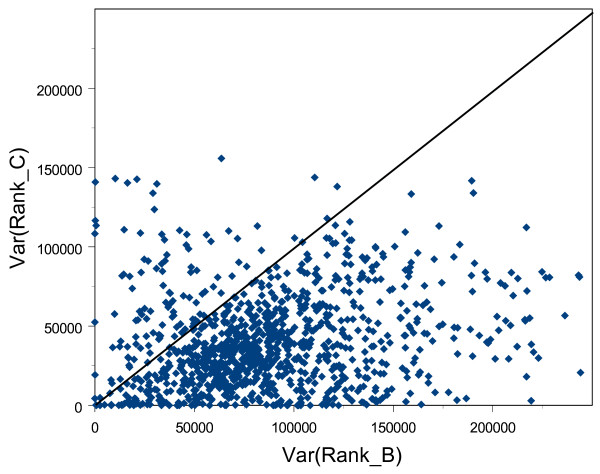
Variance of neighborhood ranks in betweenness and closeness centralities. The figure shows a scatter plot of the variance of neighborhood ranks in betweenness centrality (Var(Rank_B)) against the variance of neighborhood ranks in closeness centrality (Var(Rank_C)). The bold diagonal line represents Var(Rank_B) = Var(Rank_C). Note that most points are below the diagonal. This indicates that a node's neighborhood varies more in betweenness centrality than in closeness centrality.

Betweenness centrality not only takes account of local information (i.e. how well or richly an enzyme is connected to its neighbors), but also reflects in a semi-global manner how a node is embedded in a network. The most local measure of topological importance, degree, performs slightly less well in reflecting the phylogenetic profile of enzymes. Closeness centrality reveals an interesting insight. It is also a measure of topological importance based on how a node connects to others in the same network, but our results show that it relates poorly to phylogenetic profile. An explanation for the contrasting correlations between phylogenetic profile and the two non-local network indices may arise from the way in which closeness and betweenness centralities measure the topological importance of nodes. The closeness centralities of a node and its direct neighbors should be similar. This is because a node and its direct neighbors are only one link apart in a network. Thus, if the focal node is close to other nodes, then its direct neighbors are also likely to be close to other nodes in the same network. Conversely, if a node is far away from others, then its direct neighbors are also more likely to be far away from others. This might not be true for betweenness centrality, however, because shortest paths that pass through a given node might not pass through some of this node's immediate neighbors. Therefore, the closeness centrality of a node and its immediate neighbors will vary less than their betweenness centrality. The enzyme EC 2.7.1.69, a phosphotransferase involved in the phosphorylation of D-glucose in glycolysis, illustrates this distinction. EC 2.7.1.69 exists in 144 bacterial species, and it ranks 8th and 61st in betweenness and closeness centralities respectively. The neighborhood of EC2.7.1.69, the focal node surrounded by its immediate neighbors, produces two sub-networks. In these otherwise identical sub-networks, node sizes are proportional to different node values: in the first to closeness centrality (Figure 5a), and in the second to betweenness centrality (Figure 5b). Betweenness centrality appears to vary more between EC2.7.1.69 and its direct neighbors than does closeness centrality. Furthermore, in a similar neighborhood plot, phylogenetic profile also tends to vary more in the immediate vicinity of EC2.7.1.69 than does closeness centrality (Figure 5a, 5c). To substantiate this claim quantitatively for all nodes in the network, we ranked enzymes separately by closeness centrality, betweenness centrality and phylogenetic profile. We then defined a neighborhood for each node comprising itself and its direct neighbors, and calculated the variance of the ranks of the nodes in this neighborhood. In general, our results show that the ranks of enzymes in a given neighborhood tend to vary more for betweenness centrality than for closeness centrality (Figure 6). Furthermore, we found that the variance of ranks for phylogenetic profile in a node's neighborhood correlates with the variance of ranks for betweenness centrality (*r *= 0.25, *p *≈ 0) but not for closeness centrality (*r *= -0.01, *p *= 0.56). Our findings thus indicate that "being on many shortest paths" (i.e. high betweenness) is a quality required by enzymes with high phylogenetic profiles. They also suggest that enzymes with low betweenness centrality might be bypassed and become redundant. Such enzymes may then be prone to deletion from metabolic pathways and would therefore not occur in many bacterial species, resulting in low phylogenetic profiles. We develop this idea more fully in the next section.

### Redundancy in an enzyme network

Metabolic networks in many species have short average path lengths similar to those in a random network. This means that only a small number of reaction steps [5] connect any two metabolites. It is feasible, therefore, to envisage that nature might favor metabolic networks of a particular design that allows the rapid and reliable inter-conversion of metabolites. Consequently, an enzyme on the shortest pathway between two given enzymes should be more likely to be selected to perform a role in metabolism than enzymes on longer pathways. Our results imply this. If an enzyme is incident to many shortest paths between other enzymes (i.e. it has high betweenness centrality), then it tends to be found in many bacterial species. However, neighbors of such enzymes might have low betweenness centrality and therefore be less likely to occur in different bacterial species. In other words, if evolution favors efficient inter-conversion of metabolites, then direct neighbors of enzymes with high betweenness centrality might not be selected to play a role in metabolism and will thus become redundant.

Our rationale for investigating the issue of redundancy in the enzyme network is as follows. Imagine a linear relationship between three enzymes A, B and C, where there is a link between A and B and a link between B and C. If such a relationship is vital for cellular function in a bacterial species, then the presence of one enzyme should more or less guarantee the presence of the other two. Therefore, bacterial species having enzyme B should also have enzymes A and C. If there is a shortcut that links A and C directly, then the role of B will become redundant. Such a shortcut may provide an organism with "choices" of alternative routes of enzyme-enzyme relations, therefore some species might "select" enzymes A, B and C while some might go for enzymes A and C only. To test this idea, we determined the overlap fraction (*β*_*i*, *jk*_) between a given enzyme (*i*) and every pair of its direct neighbors (*j *and *k*) (see Methods). *β*_*i*, *jk *_is defined as the ratio of the number of bacterial species having enzymes *i*, *j *and *k *to the number of bacterial species having enzymes *j *and *k *but not *i*.

**Figure 7:**
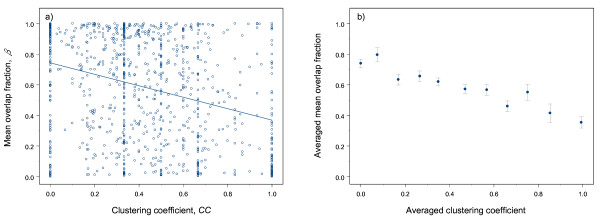
The relationship between clustering coefficient and mean overlap fraction. (a) Scatter plot of clustering coefficient (*CC*) against mean overlap fraction (*β*). (b) Relationships between averaged clustering coefficient and averaged mean overlap fraction. In (b), enzymes are divided into 11 groups according to their *CC*. The first group includes enzymes with *CC *exactly equal to zero. The second group includes enzymes whose *CC *fall within the interval (0, 0.1], the third group is for enzymes with *CC *in the interval (0.1, 0.2], and so on; the last group includes enzymes with *CC *in the interval (0.9, 1]. In (b), the plot is obtained by calculating the averaged clustering coefficient and averaged mean overlap fraction for each group, and the vertical bars are standard errors.

We speculate that if there are many direct connections between the immediate neighbors of an enzyme, then this enzyme might become redundant. The *mean *overlap fraction (see Methods) for such an enzyme should then be lower than that for enzymes with sparsely-connected neighborhoods. An appropriate network statistic that quantifies the extent of interconnection between direct neighbors of a given node is the clustering coefficient (*CC*) [23] (see Methods). We calculated the mean overlap fraction and the clustering coefficient for each enzyme and found that these quantities are negatively correlated for our enzyme network (*r*_*β*, *CC *_= -0.32, *p *≈ 0, Figure 7a). To see the relationship more clearly, we grouped enzymes according to their *CC *values, and for each group we calculated the *averaged *mean overlap fraction and the *averaged *CC. We found a more evident relationship between the mean overlap fraction and the clustering coefficient (*r*_*CC*, *β *_= -0.97, *p *= 0.002, Figure 7b). This demonstrates that enzymes in a densely-connected neighborhood (i.e. high *CC*) tend to have low overlap fractions. Moreover, we found that phylogenetic profile is also negatively correlated with the clustering coefficient (*r*_*F*, *CC *_= -0.16, *p *≈ 0) and positively with the mean overlap fraction (*r*_*F*, *β *_= 0.77, *p *≈ 0). Thus, there is evidence to support the idea that frequently-occurring enzymes are probably embedded in a sparsely-connected neighborhood and are not so redundant in the enzyme network. Furthermore, we found that betweenness centrality is negatively correlated with clustering coefficient (*r*_*B*, *CC *_= -0.48, *p *≈ 0) and positively with mean overlap fraction (*r*_***B***, *β *_= 0.23, *p *≈ 0). In contrast, closeness centrality shows little correlation with either of these quantities (*r*_*C*, *CC *_= -0.075, *p *= 0.019; *r*_*C*, *β *_= 0.12, *p *≈ 0). Therefore, enzymes on many shortest paths tend to be located in poorly-interconnected neighborhoods; and enzymes in well-interconnected neighborhoods are unlikely to be on many of the shortest paths because there are bypasses via interconnected neighbors. Consequently, enzymes that have fewer bypasses around them (via interconnected neighbors) are essential to ensure the proper functioning of sequences of metabolic reactions that involve their direct neighbors. This, in turn, might result in the co-occurrence of such enzymes and their direct neighbors in the same bacterial species.

### Factors influencing the correlations between phylogenetic profile and topological importance

From our findings, we have identified a link between the topological properties of enzymes in a network and the frequency with which these enzymes occur among bacterial species. There are correlations between phylogenetic profile and different measures of topological importance. Our study was based on an enzyme network constructed from all known enzyme-enzyme relations in 288 bacterial species, but the results might be changed by so far undiscovered enzyme-enzyme relations as well as by unexamined bacterial species. Many bacteria are obligate intracellular parasites or symbionts that can acquire enzymes and metabolites, such as those involved in amino acid metabolism, directly from their hosts [15]. Therefore, whether a bacterial species has a particular enzyme also depends on the environment in which it lives. There are other factors that explain or influence the links between phylogenetic profile of enzymes and different measures of topological importance, and we discuss them next.

Light et al. [15] suggest preferential attachment as a mechanism for the evolution of metabolic and enzyme networks. Preferential attachment means that new nodes attach to a growing network by connecting to nodes with existing high connectivity. Nodes with high connectivity are therefore often those that have been in the network for a very long time. Thus, enzymes appearing in the early stages of evolution tend to be found more frequently in different organisms (e.g. those involved in glycolysis) and that much of the metabolism of current species is based on the products of those enzymes [15]. This implies that evolutionarily early enzymes tend to have more connectivity to other enzymes or metabolites [15]. This is consistent with our findings that enzymes with high phylogenetic profile tend to have high degree. An example is pyruvate kinase, which appears in several domains of organisms (eukaryotes, bacteria and archaea); its product, pyruvate, is an important metabolite for several pathways. In our enzyme network, 224 out of 288 bacterial species have pyruvate kinase; it is the third most well-connected enzyme with a degree of 54.

There are two models for the evolution of enzymes. The first is the retrograde model, in which new enzymes evolve from older and related ones in response to the depletion of substrates in the environment [24]. The second is the patchwork model, which states that evolutionarily early enzyme tend to have broad specificity and so catalyze reactions with many different metabolites [25]. Both models can account for different aspects of metabolic evolution, and the patchwork model has recently been discussed as a major driver of enzyme evolution [14]. Patchwork models suggest that genes coding for enzymes duplicate or mutate over time to produce different variants with specific catalytic activities. Thus, ancient enzymes in different organisms tend to have more connectivity than those more recently evolved. However, degree is not always a good indicator of how many bacterial species have a particular enzyme. Bacteria often adjust their metabolism depending on their environments and acquire new enzymes via horizontal gene transfer. Light et al. [15] suggest that enzymes obtained via horizontal gene transfer are more likely to be retained by an organism if they are in a central position or are connected to several parts of a metabolic network. Thus, some high degree enzymes in a network may have been acquired recently via horizontal gene transfer, not from the evolution within a phylogeny [15]. High degree enzymes acquired via horizontal gene transfer might therefore appear in fewer species than those evolved within a phylogeny. This might in turn explain why high degree enzymes are not necessarily the most frequently-occurring among bacterial species (i.e. high phylogenetic profiles). An example is the enzyme involved in lactose utilization in *Escherichia coli. Salmonella enterica *and *Escherichia coli *are closely-related species, the latter having evolved from the former. One difference between them is that *E. coli *can utilize lactose whereas *S. enterica *cannot. Lawrence and Ochman [26] suggest that the *lac *gene that confers the ability to utilize lactose on *E. coli *was probably acquired via horizontal gene transfer. From the KEGG database, we found that *lac *codes for the enzyme beta-galactosidase, which is indeed present in *E. coli *but absent from *S. enterica*. Our results show that beta galactosidase ranks 194 by degree and 54 by betweenness centrality out of 1081 enzymes (i.e. it occupies a reasonably central position in the enzyme network), though it is found in only 64 out of 288 species.

Enzymes with many connections are more likely to be on the shortest paths between any other two enzymes. Therefore, enzymes with higher betweenness also tend to exist in many different bacterial species. It has been suggested that metabolic networks are organized compactly such that one metabolite can be converted to another in just a few reaction steps [5]. Therefore, we expect frequently-occurring enzymes to be those that appear on all shortest paths between any two other enzymes in a network. Our results show this correlation, though it is far from perfect. Flux balance analysis [27], which concerns the dynamics and stoichiometry of metabolic reactions, has shown that the metabolism of an organism does not necessarily follow the shortest paths in order to optimize its metabolic output. Instead of converting a metabolite into another in one single step, several metabolic pathways are known to comprise step-by-step changes of one metabolite into another, involving many intermediates that are precursors for other pathways [1]. An example is the citric acid cycle. Although citrate can be converted directly to oxaloacetate by the enzyme citrase, cells harbor a chain of successive reactions converting citrate into oxalacetate via several intermediate metabolites. One of these intermediates is 2-oxoglutarate which serves as a precursor in lysine biosynthesis. The enzyme that produces it, isocitrate dehydrogenase, is found in more bacterial species than citrase although it has a lower betweenness centrality. Therefore, enzymes involved in longer metabolic pathways can be as important as those on the shortest ones. Furthermore, metabolic networks often contain alternative pathways for interconverting metabolites [28]. This characteristic makes metabolic networks robust against errors, because the disruption due to the absence of one enzyme can be compensated by others [29]. Using elementary-mode analysis and experimental data, Stelling et al. [29] successfully predicted that an organism can still survive when an enzyme is removed from a metabolic network, as long as there are other routes that ensure undisrupted conversions of substrates into the final products that contribute to cellular growth. Thus, characteristics such as the robustness and redundancy of a metabolic network can provide opportunities for organisms to survive when there are faults in their enzyme-encoding genes. It is therefore possible that some enzymes, although not so important in terms of betweenness centrality (by not being on many of the shortest paths between any two enzymes), are still to be found in many different bacterial species where they ensure continued function despite faults and damages in their metabolic networks. Although we did not investigate the issue of robustness of our enzyme network against errors or nodal deletions, we found that it resembles a scale-free network with a power-law distribution; and a feature of scale-free networks is a tendency not to disintegrate into isolated parts when there are accidental nodal failures [3,8].

Finally, our results suggest that there is little relationship between phylogenetic profile and closeness centrality. The weakness of this relationship may reflect network modularity. Metabolic networks consist of functional modules such as carbohydrate metabolism, amino acid synthesis and energy metabolism. Some functional modules are located more centrally than others [2]. The consequence is that some enzymes occupy a less central position in the network despite the importance of the functional module to which they belong. Thus, closeness centrality might be a poor predictor of phylogenetic profile. An example is the enzyme DXP-synthase, which converts D-glyceraldehyde 3-phosphate and pyruvate a into 1-deoxy-D-xylulose 5-phosphate in steroid biosynthesis. Steroid biosynthesis is a part of lipid metabolism located less centrally in the metabolic network. This results in the enzyme DXP-synthase being distant from others in the enzyme network despite being found in more than half of all bacterial species (183 out of 288) on the KEGG database.

### The effect of sampling bias on the correlation between phylogenetic profile and measures of topological importance

In the KEGG database, many bacterial genera such as *Escherichia *and *Streptococcus *are represented by many species. This might introduce bias into the phylogenetic profile of different enzymes, since some genera are more highly represented than others. To investigate the effect of such a bias on our results, we determined the phylogenetic profile using information on the genus level, and tested the correlations between the revised phylogenetic profile and each measure of topological importance. Again, the *F*-*B *and *F*-*D *correlations were positive and that of *F*-*C *slightly negative: the strongest correlation is between *F *and *B *(*r*_*F*, *B *_= 0.36, *p *≈ 0), followed by that between *F *and *D *(*r*_*F*, *D *_= 0.31, *p *≈ 0) and lastly, that between *F *and *C *(*r*_*F*, *C *_= -0.07, *p *= 0.0285). Therefore, sampling bias in the phylogenetic profile does not have a significant effect on the results.

We also investigated the effect of the presence of eukaryotic species on the relationship between phylogenetic profile and each measure of topological importance. There are 32 eukaryotic species on the KEGG database, and we added all enzyme-enzyme relations found in them to the original bacterial enzyme network and then determined the phylogenetic profile for each enzyme. Again, we found the strongest correlation between *F *and *B *(*r*_*F*, *B *_= 0.38, *p *≈ 0), followed by that between *F *and *D *(*r*_*F*, *D *_= 0.34, *p *≈ 0), with the correlation between *F *and *C *being relatively weak (*r*_*F*, *C *_= -0.097, *p *= 0.0005). Since differences between bacterial and eukaryotic metabolism can be great, this consistency in our results is surprising. It could be due to the relatively small number of eukaryotic species involved in our study; their presence may not have changed the results qualitatively.

### Comparison of enzyme networks from different databases

The enzyme network we constructed from the KEGG database consists of enzyme-enzyme relations defined by successive reactions that can be mapped to specific metabolic pathways. Enzymes are involved in biochemical reactions, and an alternative enzyme network can also be constructed if a link between two enzymes is defined when compounds are shared between their respective reactions [10]. For convenience, we refer such an enzyme network as an enzyme chemical compatibility network [10]. Such a network could contain links that do not exist in real life, or links that do exist but are yet to be identified. Co-factors are molecules and ions that are required for the proper functioning of enzymes, and studies on genetic perturbation in yeast have demonstrated their functional importance in cellular metabolism [9]. Cofactors such as NAD, ATP and water take part in many biochemical reactions, so enzymes involved in different parts of a metabolic network may be brought close to each other resulting in a different type of enzyme network via those cofactor-mediated relationships. Enzyme-enzyme relations brought about via cofactor mediation are not included in the KEGG database. The resulting enzyme network without cofactors is, in essence, based on reactions that form the carbon and nitrogen-flow skeleton in metabolism. In this section, we will investigate whether our findings about the relationships between phylogenetic profile and each measure of topological importance still hold for an enzyme chemical compatibility network where cofactor-mediated relationships are also considered.

To construct an enzyme chemical compatibility network, we extracted information on bacterial species from the BioCyc database [18]. An enzyme-enzyme relation is defined between enzymes A and B if at least one of the compounds in the reaction catalyzed by A is also a compound in the reaction catalyzed by B. As before, we also determined the phylogenetic profile of enzymes. We found the strongest correlation between *F *and *B *(*r*_*F*, *B *_= 0.14, *p *≈ 0), though it was not as strong as those obtained using the KEGG database. Furthermore, we found no statistically significant correlation between *F *and *D *(*r*_*F*, *D *_= -0.008, *p *= 0.74), and as before, no correlation between *F *and *C *(*r*_*F*, *C *_= -0.004, *p *= 0.86). This was expected since the enzyme chemical compatibility network constructed here also includes enzyme-enzyme relations mediated by cofactors. This is because cofactors participate in many different biochemical reactions, making enzymes in such a network well-connected to each other. This homogenizes the topological importance of different enzymes in a network, decreasing the correlations between the phylogenetic profile and the three measures of topological importance. Cofactors are difficult to define, nonetheless, we next removed some common ones such as NAD, ATP, water, inorganic compounds and ions from the analysis and found better correlations between phylogenetic profile and the measures of topological importance (*r*_*F*, *B *_= 0.21, *p *≈ 0; *r*_*F*, *D *_= 0.07, *p *= 0.003; *r*_*F*, *C *_= -0.1, *p *≈ 0). We speculate that if we had removed all the cofactors and all the enzyme-enzyme relations that cannot be mapped to metabolic pathways, then the results would have been similar to those obtained using the KEGG database. To test our intuition, we performed further correlation tests between phylogenetic profile obtained from the BioCyc database and each measure of topological importance determined from the enzyme network constructed using the KEGG database (since the original enzyme network only has enzyme-enzyme relations that can be mapped to metabolic pathways and does not consider cofactor mediation). We found that the correlations of phylogenetic profile with degree and betweenness centrality were more evident than with closeness centrality (*r*_*F*, *D *_= 0.29, *p *≈ 0; *r*_*F*, *B *_= 0.26, *p *≈ 0; *r*_*F*, *C *_= -0.07 *p *= 0.022).

We also constructed an enzyme network for a well-studied bacterial species, *Escherichia coli *K12, from the KEGG database, and its chemical compatibility counterpart from the BioCyc database. The network constructed from the KEGG database has 609 nodes and 1392 links (an average of 2 links per node), and its counterpart from the BioCyc database has 729 enzymes and 51630 links (an average of 70 links per node). The much higher number of links per node in the enzyme chemical compatibility network is due to the presence of cofactors and nonexistent enzyme-enzyme relations. The degree or connectivity distribution of the enzyme network from the KEGG database can be fitted with a power law distribution with an estimated exponent *γ *= 1.55 (*r*^2 ^= 0.75). As for the enzyme chemical compatibility network, its degree distribution was poorly fitted by a power law (*γ *= 0.24, *r*^2 ^= 0.11), but when some common cofactors were removed from the analysis the fit improved (*γ *= 0.73, *r*^2 ^= 0.46).

Co-factors are essential components in many enzyme-catalytic reactions; and the same co-factors can participate in many different reactions. They can thus be considered to bring together enzymes in different metabolic pathways. Including co-factors in our analysis also produces a highly connected enzyme network where there is little difference in the topological importance between nodes. When co-factors are excluded, we see an enzyme network with a connectivity structure similar to the scale-free networks. All in all, our findings suggest that the connectivity structure of an enzyme network and the relationship between phylogenetic profile and measures of topological importance are profoundly dependent on the presence of cofactors. Whether or not to include co-factors in studies of similar types is open to debate as a chemical compatibility network is bound to include enzyme-enzyme relations that cannot physically occur within cells, and an enzyme-network without co-factors might miss some potential links. We suggest that future studies should investigate both cases with and without co-factors as reality is likely to fall somewhere in between.

### The effect of missing enzymes on the correlation between phylogenetic profile and topological importance

A limitation of our analysis is that only a few bacterial species in the KEGG database are fully annotated. Therefore, the phylogenetic profile obtained might be biased, since the database might lack some enzymes for some species. We pooled the data from the KEGG and the BioCyc databases and re-determined the phylogenetic profile for each enzyme. For a given enzyme, this revised profile will include bacterial species absent from the former database but present in the latter. We tested the correlations between this revised phylogenetic profile and each measure of topological importance calculated from the enzyme network constructed from the KEGG database. We found that the revised phylogenetic profile correlated more evidently with degree and betweenness centrality than with closeness centrality (*r*_*F*, *D *_= 0.33, *p *≈ 0; *r*_*F*, *B *_= 0.34, *p *≈ 0; *r*_*F*, *C *_= -0.08, *p *= 0.0102). Therefore, including bacterial species that might be absent from the KEGG database in the phylogenetic profile has little effect on our results. However, we are also aware that even in the pooled dataset, the phylogenetic profile for a given enzyme will not include all the bacterial species that contain it.

## Conclusion

In this study, we have established a link between different measures of topological importance and the likelihood that an enzyme will occur in different bacterial species. Our results suggest that betweenness centrality and degree predict the phylogenetic profile of an enzyme better than does closeness centrality. Our findings here are based on simple assumptions and many biological details have been ignored. Among these are the obvious ones that enzyme relationships are often directional and that metabolism works dynamically [30]. Furthermore, some parts of the network only exist under certain conditions because prokaryotic metabolism is characteristically determined by the environment [31]. Nevertheless, simple topological properties are still informative about the probable phylogenetic profile of an enzyme. Enzymes that have high connectivity, or play central and less redundant roles in the conversion of metabolites, are topologically more important and should occur in the majority of bacterial species.

## Methods

### Construction of the enzyme network

We constructed an enzyme network by combining information about all enzyme-catalyzed metabolic reactions in all 288 bacterial species in the KEGG database for 29 November 2005 [2]. This database is currently the best available for examining metabolic pathways. Bacteria were chosen for three reasons. First, bacterial metabolism is reasonably well understood and this allows us to identify the roles of enzymes more reliably. Second, bacteria are the largest phylogenetically limited group of species in the database, allowing good estimates to be made of the phylogenetic profile and overall topological positions of individual enzymes. Finally, limiting the investigation to a single group of organisms removes the confusion that might arise if representatives of several major organism types were examined, since each major group is likely to have metabolic characteristics peculiar to itself.

From the KEGG database, we first determined the number of different enzymes (identified by EC numbers) occurring in the different bacterial species. The number of species with a given enzyme was also determined, and we defined this number as the phylogenetic profile *F*_*i *_of enzyme *i *[17]. We defined an undirected link between two enzymes that participate in two successive reactions such that the product of one is the substrate of the other (Figure 1). The network thus constructed contains all known enzyme-enzyme relations for all bacterial species in the KEGG database.

We also extracted information on bacterial species from the BioCyc database [18] to construct an enzyme chemical compatibility network. As above, we also determined the phylogenetic profile of each enzyme. What is different here is the definition of an enzyme-enzyme relation. Here, an enzyme-enzyme relation is defined between enzymes A and B if at least one of the compounds in the reaction catalyzed by A is also a compound in the reaction catalyzed by B. Such an enzyme chemical compatibility network may contain links that cannot be mapped to metabolic pathways, or links that do exist but are yet to be identified.

### Expected phylogenetic profiles of enzymes

To test the null hypothesis that enzymes are randomly distributed among bacterial species we compared the expected rank abundance distribution with that observed in the data (Figure 3). For the expected distribution, we define *ψ*_*ij *_as the occurrence of enzyme *i *in species *j*, where *i *∈ Δ = {1,2,3,...1081} because there are 1081 enzymes and *j *∈ Γ = {1,2,3,...288} because there are 288 bacterial species. Summing the occurrences of different enzymes gives a total of 82171 occurrences (i.e. *ψ*_*ij*_). For each *ψ*_*ij*_, *i *and *j *were sampled randomly from Δ and Γ respectively so that each combination of *ij *was unique. We then counted how many times enzyme *i *occurs (*ψ*_*ij*_) and thus determined its hypothetical phylogenetic profile (*H*_*i*_). The values of *H*_*i *_were then ranked to give the expected rank distribution. This procedure was simulated 1000 times, so the expected phylogenetic profile of each rank position is the average of the 1000 simulations.

### Topological measures of importance

Centrality is a network measure of nodal importance quantifying how prominent a node is relative to others [19]. We employed three simple indices with different emphases; together, they provide the greatest amount of node-specific information [19-22]. Degree (*D*_*i*_) is the number of direct neighbors of a given node *i*. Closeness centrality (*C*_*i*_) measures how close a node *i *is to all others in the same network [20,21]:

Ci=∑j=1,i≠jNdij,
 MathType@MTEF@5@5@+=feaafiart1ev1aaatCvAUfKttLearuWrP9MDH5MBPbIqV92AaeXatLxBI9gBaebbnrfifHhDYfgasaacH8akY=wiFfYdH8Gipec8Eeeu0xXdbba9frFj0=OqFfea0dXdd9vqai=hGuQ8kuc9pgc9s8qqaq=dirpe0xb9q8qiLsFr0=vr0=vr0dc8meaabaqaciaacaGaaeqabaqabeGadaaakeaacqWGdbWqdaWgaaWcbaGaemyAaKgabeaakiabg2da9maaqahabaGaemizaq2aaSbaaSqaaiabdMgaPjabdQgaQbqabaaabaGaemOAaOMaeyypa0JaeGymaeJaeiilaWIaemyAaKMaeyiyIKRaemOAaOgabaGaemOta4eaniabggHiLdGccqGGSaalaaa@417F@

where *d*_*ij *_is the shortest distance between nodes *i *and *j*, and *N *is the number of nodes in the network. Betweenness centrality (*B*_*i*_) measures how frequently a node *i *is incident to all shortest paths between two other nodes in the same network [20,22]:

Bi=∑j>kgjk(i)/gjk,
 MathType@MTEF@5@5@+=feaafiart1ev1aaatCvAUfKttLearuWrP9MDH5MBPbIqV92AaeXatLxBI9gBaebbnrfifHhDYfgasaacH8akY=wiFfYdH8Gipec8Eeeu0xXdbba9frFj0=OqFfea0dXdd9vqai=hGuQ8kuc9pgc9s8qqaq=dirpe0xb9q8qiLsFr0=vr0=vr0dc8meaabaqaciaacaGaaeqabaqabeGadaaakeaacqWGcbGqdaWgaaWcbaGaemyAaKgabeaakiabg2da9maaqafabaGaem4zaC2aaSbaaSqaaiabdQgaQjabdUgaRbqabaGccqGGOaakcqWGPbqAcqGGPaqkcqGGVaWlcqWGNbWzdaWgaaWcbaGaemOAaOMaem4AaSgabeaaaeaacqWGQbGAcqGH+aGpcqWGRbWAaeqaniabggHiLdGccqGGSaalaaa@4391@

where *i *≠ *j*, *k*; *g*_*jk *_is the number of equally shortest paths between nodes *j *and *k*; and *g*_*jk*_(*i*) is the number of these shortest paths to which node *i *is incident.

A node with high *D*_*i *_might be important since it has many direct connections with others in the same network. A node with small *C*_*i *_might also be important simply because its influence can reach others rapidly and efficiently. Nodes with high *B*_*i *_are important because they mediate many interactions between other nodes. We examined the relationships between the phylogenetic profile and each of the three measures of topological importance by calculating their Spearman rank correlation coefficients.

### Overlap fractions

The overlap fraction (*β*_*i*, *jk*_) between a given enzyme *i *and one pair of its direct neighbors *j *and *k *is defined as the ratio of the number of bacterial species having enzymes *i*, *j *and *k *to the number of bacterial species having enzymes *j *and *k *but not *i*.

If enzyme *i *has *M*_*i *_direct neighbors, then there will be (*M*_*i*_^2 ^- *M*_*i*_)/2 neighbor pairs and therefore (*M*_*i*_^2 ^- *M*_*i*_)/2 overlap fractions. Thus, for a given enzyme *i*, we define *β*_*i *_as the mean overlap fraction:

βi=(∑j<kβi,ij)/Pi,
 MathType@MTEF@5@5@+=feaafiart1ev1aaatCvAUfKttLearuWrP9MDH5MBPbIqV92AaeXatLxBI9gBaebbnrfifHhDYfgasaacH8akY=wiFfYdH8Gipec8Eeeu0xXdbba9frFj0=OqFfea0dXdd9vqai=hGuQ8kuc9pgc9s8qqaq=dirpe0xb9q8qiLsFr0=vr0=vr0dc8meaabaqaciaacaGaaeqabaqabeGadaaakeaaiiGacqWFYoGydaWgaaWcbaGaemyAaKgabeaakiabg2da9maabmaabaWaaabuaeaacqWFYoGydaWgaaWcbaGaemyAaKMaeiilaWYaaSbaaWqaaiabdMgaPjabdQgaQbqabaaaleqaaaqaaiabdQgaQjabgYda8iabdUgaRbqab0GaeyyeIuoaaOGaayjkaiaawMcaaiabc+caViabdcfaqnaaBaaaleaacqWGPbqAaeqaaOGaeiilaWcaaa@43C9@

where *P*_*i *_is the number of neighbor pairs of enzyme *i*.

### Clustering coefficient

The clustering coefficient of a node *i*(*CC*_*i*_) is defined as [23]:

*CC*_*i *_= *Q*_*i*_/*R*_*i*_,

where *Q*_*i *_is the number of existing links between direct neighbors of node *i*, and *R*_*i *_is the number of possible links between those direct neighbors. *R*_*i *_is defined as (*M*_*i*_^2 ^- *M*_*i*_)/2, where *M*_*i *_is the number of direct neighbors of node *i*. Thus, *CC*_*i *_measures how densely connected a node's direct neighbors are. If *CC*_*i *_= 1 then all its direct neighbors are connected to each other; if *CC*_*i *_= 0 then none of its direct neighbors are connected to each other.

### Statistical test

Throughout the paper, the Spearman rank correlation test was used to determine the correlation between two variables *x *and *y *and its statistical significance. The null hypothesis is that x and y are uncorrelated (i.e. their Spearman coefficient of rank correlation is 0), and the p-value is an estimate of the probability that the null hypothesis were true. The statistical tests were performed using S-Plus.

## Authors' contributions

WCL and HTY conceived the study; WHL extracted data from the KEGG database; WCL, AJD and FJ carried out the analysis; WCL, AJD, FJ and MJH prepared the manuscript. All authors read and approved the final manuscript.
